# Targeting Innate Immunity to Treat Cancer

**DOI:** 10.3390/cancers12102723

**Published:** 2020-09-23

**Authors:** Matthew Austin, Harriet Kluger

**Affiliations:** Yale Cancer Center, Yale School of Medicine, 333 Cedar St, WWW211B, New Haven, CT 06520, USA; matthew.austin@yale.edu

In recent years, it has become clear that the immune system plays a critical role in rejecting malignant cells. Through the complex interplay between multiple cell types from both the adaptive and innate immune systems, immune cells are able to identify and destroy tumor cells. Multiple mechanisms of escape from immune surveillance have been characterized and are being harnessed for therapeutic benefit. Proposed mechanisms include, but are not limited to, alteration of surface antigens [1,2], down-regulation of necessary components for antigen presentation [3], secretion of anti-inflammatory cytokines by tumor cells or cells in the tumor micro-environment [4], and upregulation of expression of immune inhibitory molecules [5]. Drugs that target these immune-evasion mechanisms and successfully re-invigorate the immune response include inhibitors of PD-1, its ligand PDL-1, and CTLA-4, all of which have dramatically revolutionized cancer care [6,7,8]. Other classes of immune therapies that have been approved by the food and drug administration include CAR-T and natural killer (NK) cellular therapies [9], cytokine therapies [10], oncolytic viruses [11] and dendritic cell therapies, such as sipuleucel-T [12]. Immune checkpoint inhibitors, which are thought to primarily work by activation of cytotoxic T cells [13], are the most widely used; however, they are only active in a subset of cancer patients.

To overcome resistance to therapies that manipulate adaptive immunity, pre-clinical and clinical studies are also focusing on the critical role played by innate immune cells in tumor rejection. The innate immune system recognizes pathogen-associated molecular patterns present on microbes and virus-infected cells, but not in otherwise healthy host tissue. The innate immune response relies on natural physical barriers, the complement system, inflammation, and several key cell types including mast cells, macrophages, monocytes, neutrophils, basophils, dendritic cells and natural killer (NK) cells. Macrophages and neutrophils are primarily responsible for phagocytosing pathogens, releasing pro-inflammatory cytokines and chemokines, which in part, upregulate adhesion molecules on endothelial cells to induce leukocyte extravasation. Dendritic cells serve as a critical bridge between the innate and adaptive immune response by generating and presenting peptide antigens to T cells. NK cells function in a manner very similar to cytotoxic T cells but lack rearranging receptors and depend on a complex interplay between a plethora of stimulatory and inhibitory receptors for activation. 

Innate immune cells play important roles in cancer biology. Tumor-associated macrophages have functionally diverse phenotypes [14], and polarization of macrophages toward a pro-inflammatory phenotype is being studied to treat cancer. NK cells have the potential to recognize and kill malignant cells and can be harnessed for cancer therapy [15]. Dendritic cell activation has been widely studied as an adjuvant to T cell activating therapies or as a single modality to enhance antigen presentation, such as with the use of sipuleucel-T [12]. 

In this Special Issue, we review new and emerging approaches to activate innate immunity in cancer patients and present some primary research data. These approaches are being studied alone and in combination with methods to target adaptive immunity. Both pre-clinical and clinical studies are highlighted. Finally, we discuss cutting edge technologies that can further our understanding of complex immune interactions at the cellular level. 
